# Lipidomic profiling reveals free fatty acid alterations in plasma from patients with atrial fibrillation

**DOI:** 10.1371/journal.pone.0196709

**Published:** 2018-05-03

**Authors:** Youngae Jung, Youngjin Cho, Nami Kim, Il-Young Oh, Sang Won Kang, Eue-Keun Choi, Geum-Sook Hwang

**Affiliations:** 1 Integrated Metabolomics Research Group, Western Seoul Center, Korea Basic Science Institute, Seoul, Republic of Korea; 2 Department of Life Science, Ewha Womans University, Seoul, Republic of Korea; 3 Division of Cardiology, Department of Internal Medicine, Seoul National University Bundang Hospital, Seongnam, Republic of Korea; 4 Division of Cardiology, Department of Internal Medicine, Seoul National University Hospital, Seoul, Republic of Korea; 5 Department of Chemistry & Nanoscience, Ewha Womans University, Seoul, Republic of Korea; Osaka University Graduate School of Medicine, JAPAN

## Abstract

Atrial fibrillation (AF) is the most common cardiac arrhythmia, and its incidence is increasing worldwide. One method used to restore sinus rhythm is direct current cardioversion (DCCV). Despite the high success rate of DCCV, AF typically recurs within the first 2 weeks. However, our understanding of the pathophysiology of AF recurrence, incidence, and progression are highly limited. Lipidomic profiling was applied to identify altered lipids in plasma from patients with AF using ultra-performance liquid chromatography/quadrupole time-of-flight mass spectrometry coupled with multivariate statistical analysis. Partial least-squares discriminant analysis revealed a clear separation between AF patients and healthy controls. The levels of several lipid species, including fatty acids and phospholipids, were different between AF patients and healthy controls, indicating that oxidative stress and inflammation are associated with the pathogenesis of AF. Similar patterns were also detected between recurrent and non-recurrent AF patients. These results suggest that the elevated saturated fatty acid and reduced polyunsaturated fatty acid levels in AF patients may be associated with enhanced inflammation and that free fatty acid levels may play a crucial role in the development and progression of AF.

## Introduction

Atrial fibrillation (AF) is the most common arrhythmia in clinical practice. It is associated with a high risk of stroke, hospitalization, and reduced quality of life [1]. Although it can develop from a complex interaction between various factors, its pathophysiology has not yet been elucidated [2, 3]. Direct current cardioversion (DCCV) is a method used to restore sinus rhythm by electrical shock [4, 5]. Although the acute success rate of DCCV is greater than 90%, AF recurrence is common, particularly within the first 2 weeks, reaching 40% recurrence in the first month, despite treatment with anti-arrhythmic agents [6]. A long duration of AF, a large left atrium (LA), old age, underlying comorbidities, and increased heart rate variability are related to AF recurrence after DCCV [7–9]. However, the predictive values of these clinical factors are relatively low. In addition, a better understanding of the pathophysiology of AF is essential for the discovery of new therapeutic targets. As our understanding of the mechanism of AF has improved considerably, many studies have indicated that oxidative stress and inflammation play an important role in the incidence, perpetuation, and recurrence of AF [1, 10–12].

Lipids play important roles as signaling molecules, energy sources, and structural components of biological membranes [13]. Accordingly, changes in lipids due to genetic or environmental changes can greatly influence cell function, the immune system, and inflammatory responses [14]. A lipidomics approach that identifies global changes in lipid metabolites has been effectively applied in various dysregulation-related diseases, such as obesity [13, 15] and coronary artery disease [14].

To date, only a limited number of metabolic profiling studies on AF have been conducted. These reports have described the aqueous metabolite profiles of atrial tissues from AF patients [16] or animal models [17] and serum samples from AF patients [18]. And lipid profiles including total cholesterol (TC), high-density lipoprotein cholesterol (HDL-C), low-density lipoprotein cholesterol (LDL-C), and triglycerides have been provided from patients with AF to present the association with incidence of AF [19,20]. However, to the best of our knowledge, no detailed plasma lipidome as well as free fatty acid profiling have been reported in AF patients. In the present study, global lipid profiling was conducted to identify altered lipid metabolites in plasma from AF patients using ultra-performance liquid chromatography/quadrupole time-of-flight mass spectrometry (Q-TOF MS). We also investigated the impact of lipid profiles in plasma on recurrence after successful DCCV to understand the pathophysiology of AF and to present preventive and therapeutic strategies for the occurrence and recurrence of AF.

## Methods

### Study population

Patients who underwent elective DCCV for persistent AF between August 2010 and June 2013 at Seoul National University Hospital were evaluated. This study was approved by the Institutional Review Board of Seoul National University Hospital. All patients provided informed written consent. Persistent AF was confirmed by a 12-lead electrocardiogram (ECG) and 24 h Holter monitoring. Patients who had significant mitral valvular disease, had a very large LA (>60 mm), underwent emergent cardioversion, those with end-stage renal disease, or declined to consent to the study were excluded. In total, 182 patients were eligible for this study, and 52 patients who underwent electrophysiological analysis due to suspicion of paroxysmal supraventricular tachycardia without any history of AF were prospectively enrolled as healthy controls. A total of 34 patients matched by age, sex, and body mass index (BMI) were analyzed to compare the metabolomic profiles between the AF and control groups. Metabolomics profiles were also compared between AF patients who maintained sinus rhythm and who experienced AF recurrence within 1 month after electrical cardioversion, also after matching by age, sex, BMI, and LA size.

### Electrical cardioversion protocol

All patients received more than 4 weeks of adequate anticoagulation therapy (with a target International Normalized Ratio of 2.0–3.0) before elective DCCV. Before electrical DCVC, anti-arrhythmic agents were prescribed in most patients according to guidelines described previously [21]. To exclude an intra-cardiac thrombus, transesophageal echocardiography was performed on the day of cardioversion. A light sedative was administered intravenously (midazolam, 0.05–0.2 mg/kg or etomidate, 0.1 mg/kg). A biphasic R-wave synchronized shock (ZOLL M series® ACLS Defibrillator, ZOLL Medical Corporation, Chelmsford, MA, USA) was applied via paddles on the right side of the upper sternum and the left side of the left nipple. The initial cardioversion energy was 100 J and was increased to 150 and 200 J. If AF was not terminated, self-adhesive skin electrodes (ZOLL Stat-padz®, ZOLL Medical Corporation) were applied in anterior–posterior position, using a 200 J energy shock. After successful cardioversion, all patients were monitored for at least 3–4 h before discharge. After discharge, all patients received anticoagulation therapy for more than 3 months without interruption.

Then, 1 month after successful cardioversion, patients visited the outpatient clinic for evaluation with the 12-lead ECG. AF recurrence was confirmed following 12-lead ECG and physical examination. The recurrent AF group was defined as those with documented AF on the 12-lead ECG, and the sinus rhythm group (non-recurrent AF group) was defined as those on sinus rhythm at the 1-month follow-up.

### Blood sampling

Venipuncture was performed before electrical cardioversion in AF patients. Blood samples were extracted in the documented sinus rhythm prior to electrophysiological analysis in the control group. Anticoagulated whole blood samples were centrifuged at 2500rpm/700g (Heraeus Megafuge 40R, Thermo Fisher Scientific) for 15 minutes at 4°C, and plasma sample aliquots were stored at -80°C for the subsequent analysis.

### Lipid metabolite profiling using UPLC/Q-TOF MS

Plasma samples (50 μL) were extracted using a 500 μL chloroform:methanol (2:1, v/v) solution and dried under nitrogen gas. Lipid extracts were reconstituted into a 250 μL isopropanol:acetonitrile:water (2:1:1, v/v/v) solution. Finally, 5 μL solution was injected into the UPLC/Q-TOF MS system.

Lipid metabolite profiling was performed using the Waters ACQUITY UPLC system (Waters, Milford, MA, USA) with a triple TOF 5600 Mass Spectrometer (SCIEX, Framingham, MA, USA). Separation was performed on an Acquity UPLC BEH C18 (2.1 × 100 mm) with 1.7 μm particles (Waters). Mobile phases A and B involved 10 mM ammonium acetate in an acetonitrile:water (4:6, v/v) solution and 10 mM ammonium acetate in an acetonitrile:isopropanol (1:9, v/v) solution, respectively. Samples were eluted at 0.35 mL/min for 19 min. The mass spectrometer was analyzed in the electrospray ionization positive and negative ion modes, and the mass range was set at m/z 100–1500. Accurate mass measurements for each peak were obtained with an automated calibrant delivery system (CDS) using 0.2 mL/min of positive and negative calibration solution (SCIEX) containing internal reference compounds.

Spectral data were analyzed with MarkerView software (SCIEX), which was used to identify peaks, perform the alignment, and generate peak tables of m/z and retention times. Lipid metabolites were identified using Lipid Maps (www.lipidmaps.org), the Human Metabolome Database (www.hmdb.ca), and Metlin (metlin.scrips.edu). Data were confirmed using standard samples (Avanti Polar Lipids, Alabaster, AL, USA and Sigma-Aldrich, St. Louis, MO, USA) based on retention times and MS/MS spectra.

### RT-qPCR analysis

Additional plasma samples were collected from 6 healthy controls and 6 patients with AF to measure the mRNA cytokine. Total RNA was extracted from human plasma using a miRNeasy Serum/Plasma Kit (Qiagen, Valencia, CA, USA) according to the manufacturer’s protocol. RNA concentration and quality were immediately determined using a Nanodrop 2000 (Thermo Fisher Scientific, Waltham, MA, USA). Human plasma RNA served as a template for synthesizing cDNA using the GoTaq® 1-Step RT-qPCR System according to the manufacturer’s instructions (Promega, Madison, WI, USA). Reactions were carried out using SYBR Green for 40 cycles of denaturation at 95°C for 10 s, annealing at 60°C for 30 s, and extension at 72°C for 30 s using the StepOnePlus Real-Time PCR System (Applied Biosystems, Foster City, CA, USA). qPCR was performed using the following primers: IL-1β sense (5′-TGG GAT AAC GAG GCT TAT GTG-3′) and antisense (5′-ATG GAG AAC ACC ACT TGT TGC-3′), tumor necrosis factor alpha (TNF-α) sense (5′-CTC CTA CCA GAC CAA GGT CAA C-3′) and antisense (5′-AGA CTC GGC AAA GTC GAG ATA G-3′), and β-actin sense (5′-CCA CGA AAC TAC CTT CAA CTC C-3′) and antisense (5′-GGA GCA ATG ATC TTG ATC TTC A-3′). The experiment was performed on three independent biological replicates. Gene expression was normalized to the mRNA expression level of β-actin (endogenous control). For control samples, fold changes were calculated using relative quantification.

### Statistical analysis

Multivariate analyses were conducted using SIMCA-P+ software version 12.0 (Umetrics, Umeå, Sweden). Principle component analysis (PCA) was applied to determine the intrinsic variation in the data set, and partial least squares discriminant analysis (PLS-DA) was used as a classification method. Lipid metabolites with a variable importance in the projection (VIP) score > 1 were considered to be the metabolites responsible for the differences between healthy controls and AF patients.

SPSS 15.0 (SPSS Inc., Chicago, IL, USA) was used for all statistical analyses. Mann–Whitney *U*, chi-square tests, and Fisher’s exact test were used to detect differences in the clinical characteristics and lipid metabolites between healthy controls and patients (*p* < 0.05). Spearman’s correlation coefficient was used to determine the relationships between clinical parameters and levels of free fatty acids (FFAs).

## Results

### Patient characteristics

Baseline clinical characteristics of age-, sex-, and BMI-matched AF patients and controls are shown in Table 1. There were no significant differences in the presence of comorbidities such as hypertension and diabetes mellitus, between the AF and control groups. The size of the LA measured by echocardiography was significantly larger in the AF group (AF vs. control: 47.6 ± 7.0 vs. 36.7 ± 5.9, *p* < 0.001). Anticoagulant and anti-arrhythmic agents were more frequently prescribed in patients with AF than healthy controls. The proportion of patients taking a statin or an anti-diabetic agent did not differ between groups.

**Table 1:** Clinical characteristics of healthy controls and patients with AF.

	Healthy controls (n = 34)		AF patients (n = 34)		*P* value
Age (years)	54.1	± 8.67	56.2	± 9.70	0.354
Male/Female	13	/ 21	13	/ 21	1.000
BMI (kg/m^2^)	24.1	± 3.59	24.6	± 2.90	0.497
Hypertension	8	(23.5)	11	(32.4)	0.417
Diabetes	4	(11.8)	3	(8.8)	1.000
Hypercholesterolemia	4	(11.8)	4	(11.8)	1.000
Coronary artery disease	0	(0.0)	0	(0.0)	-
Congestive heart failure	0	(0.0)	0	(0.0)	-
Cerebrovascular accident	0	(0.0)	0	(0.0)	-
LA size, mm	36.7	± 5.9	47.6	± 7.0	<0.001
LA volume, mL	36.4	± 9.0	97.0	± 32.7	<0.001
Medication					
Anticoagulant agent	0	(0.0)	33	(97.1)	<0.001
Antiplatelet agent	0	(0.0)	1	(2.9)	1.000
Anti-arrhythmic drug	0	(0.0)	33	(97.1)	<0.001
ACE inhibitor or ARB	7	(20.6)	12	(35.3)	0.177
Beta-blocker	1	(2.9)	3	(8.8)	0.614
Calcium channel blocker	4	(11.8)	4	(11.8)	1.000
Statin	3	(8.8)	4	(11.8)	1.000
Anti-diabetic drugs	2	(5.9)	1	(2.9)	1.000

Data are presented as means ± standard deviations. *P* values were calculated using the Mann–Whitney *U*, chi-square tests, and Fisher’s exact test with significance set at *p* < 0.05. Abbreviations: AF, atrial fibrillation; BMI, body mass index; LA, left atrium; ACE inhibitor, angiotensin-converting enzyme inhibitor; ARB, angiotensin receptor blocker.

### Lipid profiling of AF patients

Total ion chromatograms of lipid extracts were obtained by UPLC/Q-TOF MS in positive and negative ion modes (S1 Fig). Phosphatidylcholine (PC), phosphatidylethanolamine (PE), sphingomyelin (SM), and triglyceride (TG) were detected in the positive ion mode, and FFAs, phosphatidylinositol (PI), and phosphatidic acid (PA) were detected in the negative ion mode. Lipid species were identified by the accurate mass, isotope patterns, MS/MS fragmentation data, and relative retention time of the same species.

**Fig 1:**
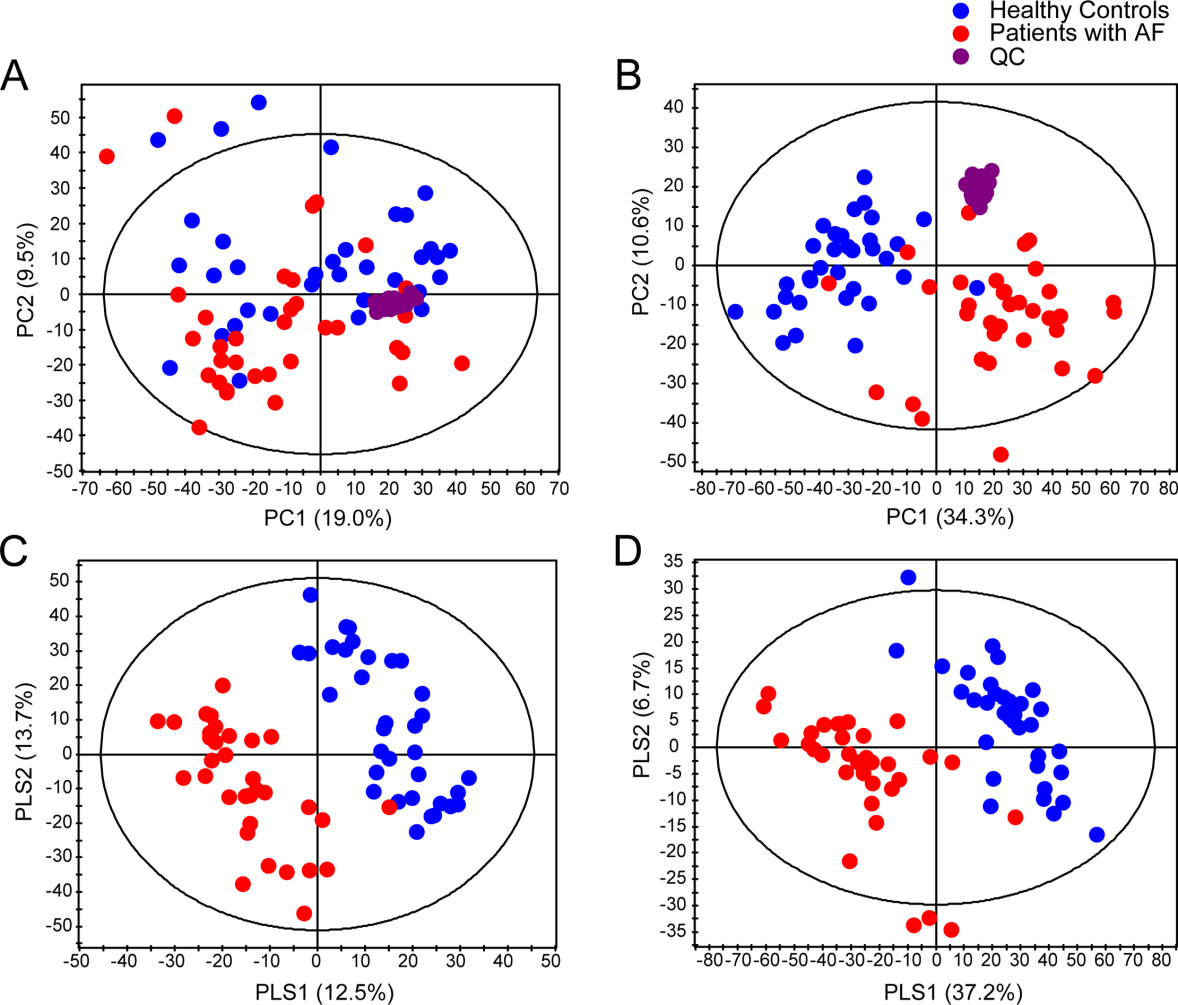
Principle component analysis (PCA) and partial least squares discriminant analysis (PLS-DA) score plots derived from the ultra-performance liquid chromatography/quadrupole time-of-flight mass spectrometry spectra of plasma samples from healthy controls and patients with AF. PCA score plots in positive (A; R2X = 0.680, Q2 = 0.456) and negative (B; R2X = 0.699, Q2 = 0.521) ion mode, and PLS-DA score plots in positive (C; R2X = 0.387, R2Y = 0.967, Q2 = 0.863) and negative (D; R2X = 0.477, R2Y = 0.934, Q2 = 0.846) ion mode. Each ellipse was given by Hotelling’s T2 (0.95).

Multivariate pattern recognition methods were applied to identify lipidomic changes in patients with AF. Principle component analysis (PCA) score plots showed better separation in the negative ion mode between patients and healthy controls than in the positive ion mode (Fig 1A and 1B). The reproducibility of metabolite signals was confirmed by quality control samples injected repeatedly between samples in the PCA score plots. According to partial least squares discriminant analysis (PLS-DA), the two groups were clearly separated in both polarity modes (positive ion mode: R2X = 0.387, R2Y = 0.967, Q2 = 0.863; negative ion mode: R2X = 0.477, R2Y = 0.934, Q2 = 0.846) (Fig 1C and 1D).

Lipid metabolites with VIP scores >1 in the PLS-DA and a *p* value < 0.05 were considered significant lipid species; a total of 33 lipids were identified (Table 2). The levels of all identified FFAs were lower in AF patients than in the controls. LysoPC, LysoPE, and PC levels were greater in AF patients than in controls when they had a relatively low degree of unsaturation; by contrast, these species were decreased in AF patients when they had a relatively high degree of unsaturation. Regarding the other lipid classes (i.e., PE, SM, TG, PA, and PI), only one lipid per class was significantly upregulated in AF patients relative to controls.

**Table 2:** Lipids responsible for the differences between healthy controls and patients with AF.

Class	Identification	Healthy controls (n = 34)	AF patients (n = 34)	VIP value	Fold change (AF/Con)	*P* value
SFA	FA 14:0	360 ± 91.1	216 ± 47.5	1.32	0.601	<0.001
	FA 16:0	4780 ± 515	3550 ± 452	1.38	0.743	<0.001
MUFA	FA 16:1	1180 ± 367	621 ± 372	1.10	0.528	<0.001
	FA 18:1	6890 ± 807	4240 ± 1160	1.39	0.616	<0.001
PUFA	FA 16:2	23.9 ± 7.61	9.37 ± 4.05	1.34	0.393	<0.001
	FA 18:2	4650 ± 636	2320 ± 815	1.51	0.498	<0.001
	FA 18:3	826 ± 311	328 ± 174	1.27	0.398	<0.001
	FA 20:2	89.8 ± 21.7	47.4 ± 12.9	1.33	0.527	<0.001
	FA 20:3	117 ± 44.9	49.3 ± 14.1	1.26	0.420	<0.001
	FA 20:4	246 ± 109	132 ± 37.4	1.00	0.539	<0.001
	FA 22:4	48.0 ± 18.0	21.9 ± 6.43	1.21	0.455	<0.001
	FA 22:5	198 ± 102	74.3 ± 32.0	1.10	0.376	<0.001
LysoPC	LysoPC 14:0	19.4 ± 4.29	26.7 ± 7.07	1.79	1.376	<0.001
	LysoPC 16:0	1720 ± 264	2210 ± 349	2.09	1.286	<0.001
	LysoPC 18:0	753 ± 157	893 ± 193	1.24	1.186	0.003
	LysoPC 18:1	331 ± 70.7	403 ± 103	1.29	1.218	0.005
	LysoPC 20:1	6.78 ± 1.86	8.09 ±1.79	1.19	1.193	0.003
	LysoPC 20:3	52.3 ± 23.9	38.3 ± 9.63	1.33	0.731	0.003
	LysoPC 22:4	2.76 ± 1.34	1.85 ± 0.597	1.45	0.670	0.001
	LysoPC 22:5	15.0 ± 9.36	11.2 ± 3.58	1.02	0.749	0.013
	LysoPC 22:6	108 ± 77.2	71.1 ± 23.6	1.13	0.658	<0.001
PC	PC 30:0	109 ± 44.6	137 ± 48.9	1.12	1.249	0.007
	PC 32:0	562 ± 112	653 ± 114	1.30	1.162	<0.001
	PC 38:3	1000 ± 291	848 ± 186	1.19	0.845	0.044
	PC 40:7	22.4 ± 5.87	19.8 ± 4.11	1.13	0.885	0.048
LysoPE	LysoPE 16:0	9.74 ± 2.03	13.0 ± 2.93	1.82	1.337	<0.001
	LysoPE 18:0	14.0 ± 2.05	17.4 ± 3.02	1.81	1.238	<0.001
	LysoPE 20:4	18.2 ± 4.60	15.1 ± 4.02	1.23	0.834	<0.001
	LysoPE 22:6	30.2 ± 11.1	23.3 ± 12.8	1.12	0.772	<0.001
PE	PE 36:4	1.88 ± 0.407	2.21 ± 0.529	1.11	1.173	0.016
SM	SM d34:2	442 ± 79.6	506 ± 89.0	1.21	1.146	0.002
PA	PA 34:2	4.08 ± 0.909	5.48 ± 1.20	1.04	1.341	<0.001
PI	PI 36:1	10.5 ± 3.24	16.6 ± 6.81	1.08	1.582	<0.001
	PI 38:5	6.77 ± 2.40	8.60 ± 2.94	1.09	1.269	0.005

Data are presented as means ± standard deviations. Intensity was divided by 10^4^. *P* values were calculated using the Mann–Whitney *U* test, with significance set at *p* < 0.05. Abbreviations: AF, atrial fibrillation; VIP, variable importance in the projection; SFA, saturated fatty acid; MUFA, monounsaturated fatty acid; PUFA, polyunsaturated fatty acid; LysoPC, lysophosphatidylcholine; PC, phosphatidylcholine; LysoPE, lysophosphatidylethanolamine; PE, phosphatidylethanolamine; SM, sphingomyelin; PA, phosphatidic acid; PI, phosphatidylinositol.

### Altered FFAs in AF patients

Although all FFAs examined were downregulated in AF patients relative to controls, other lipid species showed opposite patterns according to the degree of unsaturation. Therefore, all detectable FFAs in plasma samples were examined. The intensities and compositions of the 18 FFAs are presented in Table 3. While the intensities of C14:0 and C16:0 saturated fatty acids (SFAs) were significantly lower in AF patients, the intensities of longer SFAs, such as C18:0, C20:0, and C22:0, were significantly higher in AF patients. The levels of all monounsaturated FFAs (MUFAs) and polyunsaturated FFAs (PUFAs) were significantly lower in AF patients. The FFA composition (% of total FFAs) indicated a higher percentage of SFAs (43.7% ± 6.2% vs. 32.9% ± 3.1%, *p* < 0.001) and a lower percentage of MUFAs (33.7% ± 4.4% vs. 36.7% ± 2.6%, *p* = 0.003) and PUFAs (22.6% ± 3.9% vs. 30.5% ± 3.3%, *p* < 0.001) in AF patients than in controls.

**Table 3:** Intensities and compositions of free fatty acids (% of total free fatty acids) in plasma samples obtained from healthy controls and AF patients.

FFA	FFA intensity			FFA composition		
	Con (n = 35)	AF (n = 35)	*P* value	Con (n = 35)	AF (n = 35)	*P* value
C14:0	360 ± 91.1	216 ± 47.5	<0.001	1.61 ± 0.380	1.52 ± 0.244	0.397
C16:0	4780 ± 515	3550 ± 452	<0.001	21.5 ± 1.84	25.1 ± 2.51	<0.001
C18:0	2120 ± 275	2340 ± 341	0.004	9.63 ± 1.67	16.8 ± 3.83	<0.001
C20:0	14.7 ± 4.02	20.4 ± 7.16	<0.001	0.0671 ± 0.0216	0.149 ± 0.0653	<0.001
C22:0	11.7 ± 3.11	14.1 ± 2.54	<0.001	0.0534 ± 0.0179	0.103 ± 0.0292	<0.001
C16:1	1180 ± 367	621 ± 372	<0.001	5.22 ± 1.35	4.11 ± 1.75	0.002
C18:1	6890 ± 807	4240 ± 1160	<0.001	30.9 ± 1.94	29.1 ± 3.00	0.007
C20:1	120 ± 61.0	68.7 ± 27.9	<0.001	0.536 ± 0.262	0.471 ± 0.155	0.326
C16:2	23.9 ± 7.61	9.37 ± 4.05	<0.001	0.106 ± 0.0274	0.0634 ± 0.0179	<0.001
C18:2	4650 ± 636	2320 ± 815	<0.001	20.9 ± 2.09	15.8 ± 3.24	<0.001
C18:3	826 ± 311	328 ± 174	<0.001	3.68 ± 1.26	2.28 ± 1.25	<0.001
C18:4	26.1 ± 22.6	8.56 ± 4.01	<0.001	0.116 ± 0.0982	0.0601 ± 0.0281	<0.001
C20:2	89.8 ± 21.7	47.4 ± 12.9	<0.001	0.399 ± 0.0713	0.326 ± 0.0481	<0.001
C20:3	117 ± 44.9	49.3 ± 14.1	<0.001	0.517 ± 0.164	0.341 ± 0.0520	<0.001
C20:4	246 ± 109	132 ± 37.4	<0.001	1.08 ± 0.376	0.928 ± 0.216	0.027
C22:4	48.0 ± 18.0	21.9 ± 6.43	<0.001	0.212 ± 0.0657	0.151 ± 0.0224	<0.001
C22:5	198 ± 102	74.3 ± 32.0	<0.001	0.867 ± 0.378	0.506 ± 0.148	<0.001
C22:6	602 ± 412	307 ± 127	<0.001	2.64 ± 1.56	2.132 ± 0.827	0.198
SFA	7290 ± 689	6140 ± 637	<0.001	32.9 ± 3.12	43.7 ± 6.19	<0.001
MUFA	8190 ± 1080	4930 ± 1500	<0.001	36.7 ± 2.58	33.7 ± 4.38	0.003
PUFA	6830 ± 1200	3290 ± 1070	<0.001	30.5 ± 3.28	22.6 ± 3.90	<0.001

Data are presented as means ± standard deviations. Intensity was divided by 10^4^. *P* values were calculated using the Mann–Whitney *U* test. Abbreviations: AF, atrial fibrillation; SFA, saturated fatty acid; MUFA, monounsaturated fatty acid; PUFA, polyunsaturated fatty acid.

### Predicting the recurrence of AF after DCCV

To investigate the metabolic differences based on the recurrence of AF, the analyses described above were repeated on age-, sex-, and BMI-matched AF patients who experienced AF recurrence within 1 month after DCCV (recurrent AF group, n = 57) versus those who maintained sinus rhythm (non-recurrent AF group, n = 57). All baseline clinical characteristics were similar between the two groups (S1 Table).

**Fig 2:**
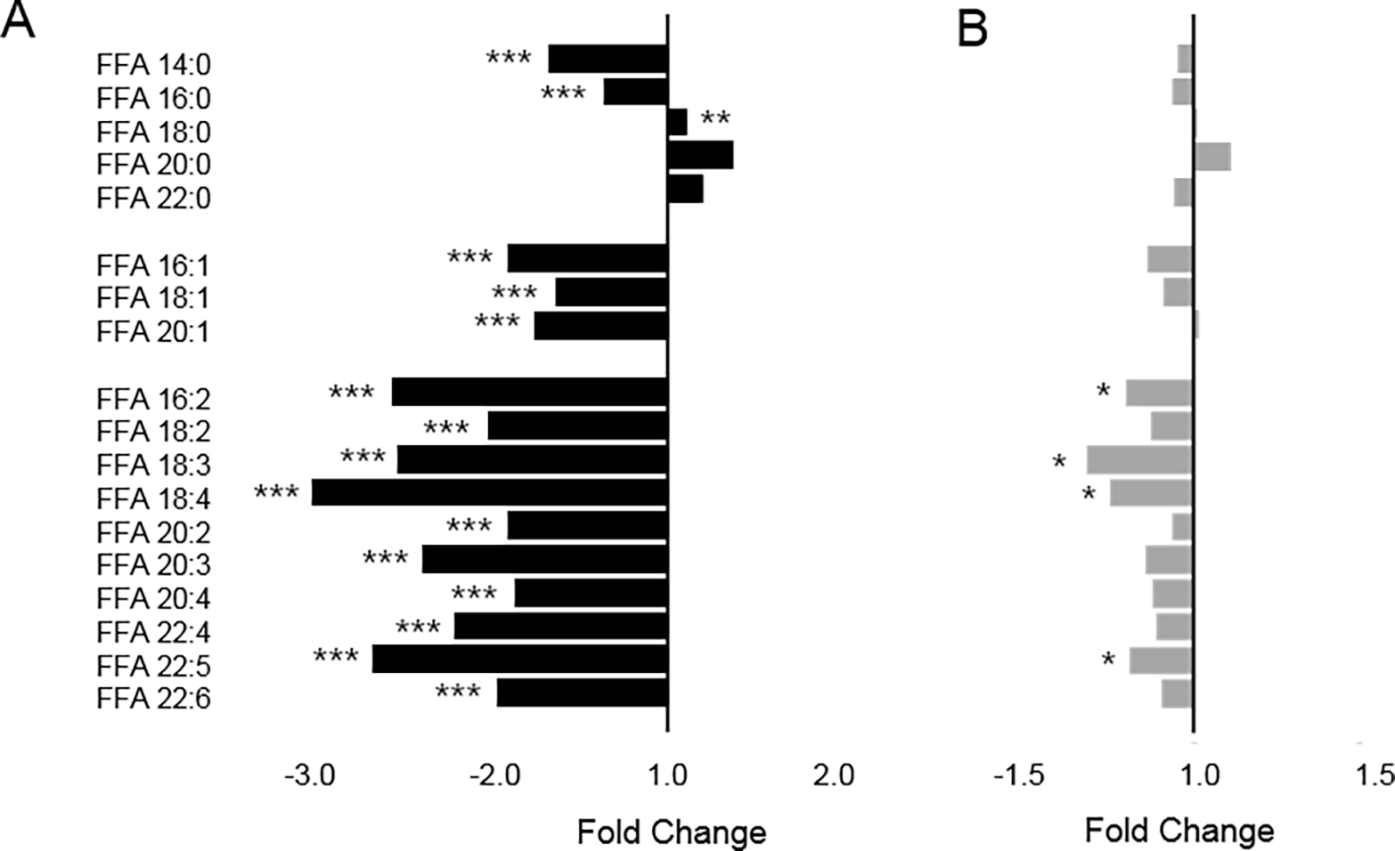
Intensities of free fatty acids in plasma samples obtained from healthy controls and AF patients and (A) and non-recurrent and recurrent AF patients (B). P values were calculated using the Mann–Whitney U test. *p < 0.05, **p < 0.01, ***p < 0.001.

**Fig 3:**
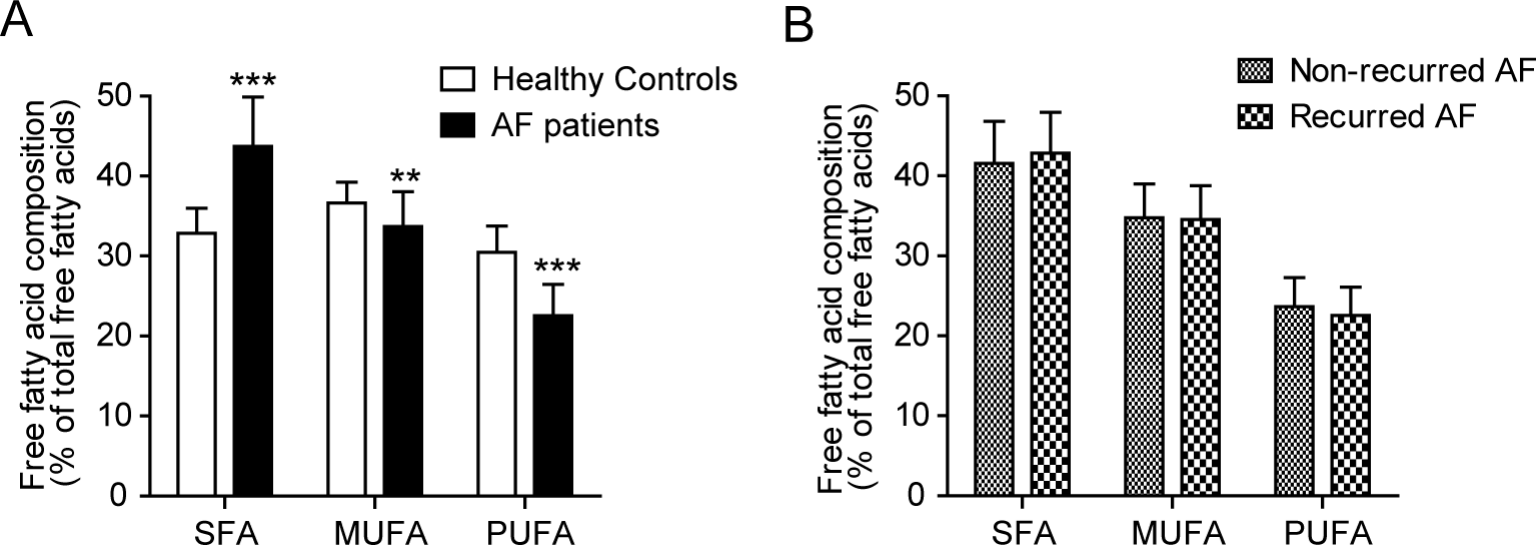
Compositions of SFAs, MUFAs, and PUFAs (% of total free fatty acids) in plasma samples obtained from healthy controls and AF patients (A) and non-recurrent and recurrent AF patients (B). Data are presented as means ± standard deviations. P values were calculated using the Mann–Whitney U test. **p < 0.01, ***p < 0.001.

Multivariate analyses on lipidomics did not indicate a definite separation according to AF recurrence (S2 Fig). The intensities and compositions of 18 FFAs were also identified, and the intensities of FA 16:2, 18:3, 18:4, and C22:5 were significantly lower in the recurrent AF group (S2 Table). Although the intensities of other FFAs were not significantly different between recurrent and non-recurrent AF patients, the pattern of changes was similar to that between the AF and control groups (Fig 2). A comparison of FFA composition (SFA, MUFA, and PUFA) also revealed a similar pattern of change (increased SFAs, and decreased MUFAs and PUFAs in recurrent AF patients), although the differences were not statistically significant (Fig 3).

### Relative mRNA levels of inflammatory cytokines

**Fig 4:**
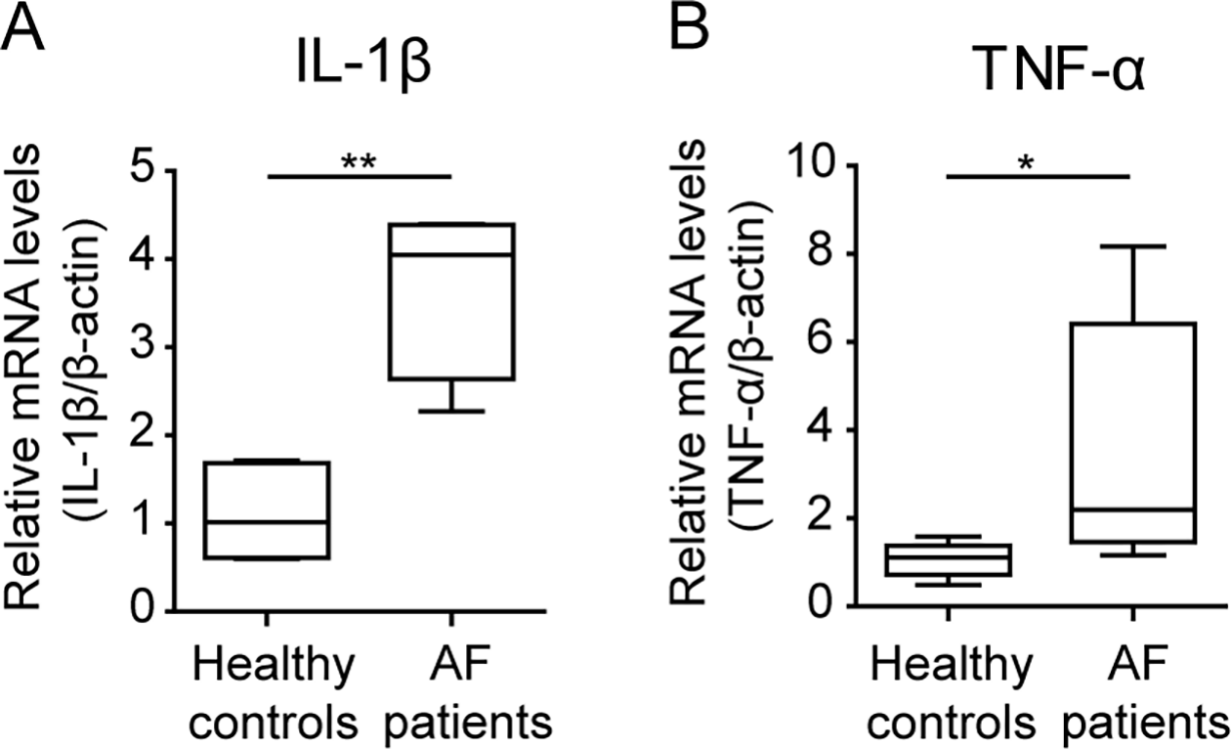
Relative mRNA levels of inflammatory cytokines in plasma samples obtained from healthy controls and AF patients. A, IL-1β (healthy controls, n = 6; AF patients, n = 4); B, TNF-α (healthy controls, n = 6; AF patients, n = 6). P values were calculated using the Mann–Whitney U test. *p<0.05, **p<0.01.

Relative mRNA expression levels of inflammatory cytokines such as interleukin (IL)-1β and tumor necrosis factor (TNF)-α were measured to confirm the inflammation condition in plasma samples of patients with AF. The expression in IL-1β and TNF-α were significantly increased in AF patients (Fig 4).

## Discussion

Lipids have a wide range of biological functions, including energy storage, membrane structure, and cell signaling, with various lipid compositions and distributions [22, 23]. Changes in lipid metabolism are closely related to disease states, and thus a comprehensive lipid analysis in biological systems can provide a better understanding of the pathogenesis of diseases and disease biomarkers for the diagnosis and prognosis of diseases, pharmaceutical discovery, and therapeutic effects [22].

We performed global lipid profiling to understand the lipid changes in AF patients. In PCA score plots, AF patients were clearly distinguished from healthy controls, and certain lipid metabolites that were altered in AF patients (i.e., FFAs, LysoPC, LysoPE, and PC) were confirmed to be responsible for this separation. In particular, we found that a characteristic change depends on the degree of unsaturation of fatty acids in the specific lipid classes, including FFAs and phospholipids. To better understand this association, we investigated all FFAs in plasma samples. In total, 18 FFAs detected in AF patients exhibited distinct differences, depending on the degree of unsaturation: SFAs were upregulated, and MUFAs and PUFAs were downregulated in AF patients relative to controls.

Although electrical cardioversion is usually successful in restoring sinus rhythm in patients with AF [4], maintaining sinus rhythm after successful cardioversion is often difficult. Old age, a large LA, the presence of other cardiac problems, and a longer duration of AF are correlated with AF recurrence [23, 24]. We investigated whether metabolic changes can predict AF recurrence after successful DCCV. For this purpose, we compared the lipidomic profiles between recurrent and non-recurrent AF patients, after matching to adjust for other potential risk factors of AF recurrence. Although 12-lead ECG has limiter power to detect paroxysmal AF, most of AF recurred after cardioversion were persistent type which 12-lead ECG could detect. The lipidomic differences between the recurrent and non-recurrent AF patients were not significant, nevertherless the intensities of FFAs showed a similar pattern to those from the comparison between AF and the control group: the SFA level in plasma was higher in the recurrent AF group, while the levels of MUFAs and PUFAs were lower in the recurrent AF group. As patients with progressed AF are more likely to experience AF recurrence after rhythm treatment [25], these results support the lipidomic differences in AF patients compared to healthy controls.

Although FFAs are a small proportion of the total fatty acids in plasma, they serve many important functions in the body [26]. FFAs released from adipose tissue by lipolysis are an important energy source and are tightly regulated in accordance with the energy needs of the body [27, 28]. FFA oxidation requires more oxygen than glycolysis, and elevated FFAs are related to cardiovascular diseases [27]. In particular, excess FFAs lead to plasma membrane damage and contribute to myocardial dysfunction and ventricular fibrillation [27, 28]. In a large prospective cohort study, the plasma concentration of total FFAs was shown to predict the future risk of AF occurrence [29]. However, not all FFAs act similarly in cardiovascular diseases: they are believed to function differently depending on their chemical structure [30–32]. Because chain length and saturation can affect the permeability, rigidity, and fluidity of the phospholipid membrane, changes in the degree of unsaturation or chain length may cause different responses for oxidative damage by reactive oxygen species [30]. The increased levels of SFAs in the AF patients may have been influenced by adipose-stimulated lipolysis [32] and thus contributed to inflammation [31]. Conversely, the decrease in PUFAs may have resulted from their degradation from reactive oxygen species [32]. PUFAs also exhibit anti-inflammatory effects by downregulating the release of pro-inflammatory cytokines [31, 33]. Moreover, the present data are consistent with a previous study in which SFAs and PUFAs were shown to play different pro- and anti-inflammatory roles with regard to inflammation [31]. This study demonstrated that FFAs, including SFAs, MUFAs, and PUFAs, are elevated or reduced in plasma samples of AF patients, supporting an important role for inflammation in the pathogenesis of AF.

Several studies have reported an association between inflammation and AF and shown elevated levels of inflammatory markers or mediators, such as C-reactive protein (CRP), TNF-α, IL-2, IL-6, and IL-8 in patients with AF [12, 34–37]. It is known that PUFAs might regulate the expression of genes related to promoting inflammation [38]. Actually, PUFA supplementation reduced mRNA levels for inflammatory cytokines including IL-1β and TNF-α in the mononuclear cells isolated from human whole blood [39], human gastric tissues [40], bovine chondrocytes [41]. Furthermore, emerging evidence suggests that inflammation plays a major role in the initiation and perpetuation of AF. In this study, CRP levels were not routinely measured in the enrolled patients. Instead, relative mRNA expression levels of inflammatory cytokines, such as IL-1β and TNF-α, which revealed that the expression of IL-1β and TNF-α was significantly upregulated in AF patients relative to controls. However, recent clinical trials reported little evidence of benefit of supplementation on incident or recurrent AF [42], therefore, further studies on the correlation between cytokines expression and lipid levels would be helpful to evaluate the association between inflammatory cytokine mRNA and lipid species. We suggest that reduced PUFAs may affect inflammatory cytokine production and the elevated expression of cytokines could be associated with the initiation and/or perpetuation of AF.

In conclusion, AF patients showed discernible lipid profiles compared to healthy controls: levels of SFA were elevated, while PUFA levels were decreased in plasma from AF patients. These changes may be associated with enhanced inflammation and pro-arrhythmic conditions, supporting the association between FFA levels and the risk of AF development and progression.

## Supporting information

S1 Table Clinical characteristics for non-recurred and recurred AF patients. (DOCX)

S2 Table Intensities and compositions of free fatty acids (% of total free fatty acids) in plasma samples in non-recurred and recurred AF patients. (DOCX)

S1 Fig Total ion chromatogram of plasma extracts obtained from QC sample. (A) Positive ion mode and (B) negative ion mode.
(TIF)

S2 Fig PCA score plots derived from the ultra-performance liquid chromatography/quadrupole time-of-flight mass spectrometry (UPLC/Q-TOF MS) spectra of plasma from non-recurred and recurred AF patients. PCA score plots in positive (A; R2X = 0.708, Q2 = 0.526) and negative ion mode (B; R2X = 0.705, Q2 = 0.509). Each ellipse was given by Hotelling’s T2 (0.95).
(TIF)
