# Supplementary material for: Lipidomic profiling reveals free fatty acid alterations in plasma from patients with atrial fibrillation
Source: PLoS One. 2018 May 3;13(5):e0196709. doi: 10.1371/journal.pone.0196709 (PMC5933795; doi:10.1371/journal.pone.0196709)
Supplement: S1 Table — (DOCX) [file pone.0196709.s001.docx]

**S1 Table. Clinical characteristics for non-recurred and recurred AF patients.**

|  | Non-recurred AF  (n=57) | | Recurred AF  (n=57) | | *p*-value |
| --- | --- | --- | --- | --- | --- |
| Age (years) | 64.4 | ± 10.2 | 63.8 | ± 9.31 | 0.509 |
| Male/Female | 39 | /18 | 39 | /18 | 1.000 |
| BMI (kg/m^2^) | 24.2 | ± 2.92 | 24.5 | ± 2.63 | 0.557 |
| Hypertension | 31 | (54.4) | 26 | (45.6) | 0.349 |
| Diabetes | 18 | (31.6) | 14 | (24.6) | 0.404 |
| Hypercholesterolemia | 30 | (52.6) | 25 | (43.9) | 0.349 |
| Coronary artery disease | 2 | (3.5) | 1 | (1.8) | 1.000 |
| Congestive heart failure | 2 | (3.5) | 3 | (5.3) | 1.000 |
| Cerebrovascular accident | 3 | (5.3) | 1 | (1.8) | 0.618 |
| LA size, mm | 50.3 | ± 6.82 | 50.4 | ± 6.59 | 0.704 |
| LA volume, mL | 98.4 | ± 37.6 | 95.0 | ± 27.9 | 0.927 |
| Medication |  |  |  |  |  |
| Anticoagulant agent | 55 | (96.5) | 53 | (93.0) | 0.679 |
| Antiplatelet agent | 7 | (12.3) | 5 | (8.8) | 0.762 |
| Antiarrhythmic drug | 50 | (87.7) | 55 | (96.5) | 0.162 |
| ACE inhibitor or ARB | 22 | (38.6) | 23 | (40.4) | 0.848 |
| Beta-blocker | 14 | (24.6) | 13 | (22.8) | 0.826 |
| Calcium channel blocker | 9 | (15.8) | 8 | (14.0) | 0.793 |
| Statin | 21 | (36.8) | 16 | (28.1) | 0.317 |
| Anti-diabetic drugs | 8 | (14.0) | 6 | (10.5) | 0.568 |

The data are presented as the mean ± SD and *p*-values were calculated from Mann-Whitney U test and Chi-square test with significance at *p*<0.05. Abbreviations: AF, atrial fibrillation; BMI, body mass index; LA, left atrial; ACE inhibitor, Anangiotensin-converting enzyme inhibitor; ARB, Angiotensin receptor blocker.
