# Supplementary material for: Lipidomic profiling reveals free fatty acid alterations in plasma from patients with atrial fibrillation
Source: PLoS One. 2018 May 3;13(5):e0196709. doi: 10.1371/journal.pone.0196709 (PMC5933795; doi:10.1371/journal.pone.0196709)
Supplement: S2 Table — (DOCX) [file pone.0196709.s002.docx]

**S2 Table. Intensities and compositions of free fatty acids (% of total free fatty acids) in plasma samples in non-recurred and recurred AF patients.**

| FFAs | FFAs intensity | | |  | FFAs composition (%) | | | |
| --- | --- | --- | --- | --- | --- | --- | --- | --- |
|  | Non-recurred AF (n=57) | Recurred AF (n=57) | *p*-value |  | Non-recurred AF (n=57) | Recurred AF (n=57) | *p*-value | |
| C14:0 | 233±73.3 | 224±43.9 | 0.980 |  | 1.45±0.313 | 1.49±0.216 | 0.112 | |
| C16:0 | 3930±749 | 3720±478 | 0.199 |  | 24.5±2.37 | 24.8±2.03 | 0.357 | |
| C18:0 | 2410±312 | 2420±413 | 0.652 |  | 15.4±3.33 | 16.3±3.37 | 0.231 | |
| C20:0 | 20.2±6.78 | 22.3±14.3 | 0.636 |  | 0.132±0.0547 | 0.157±0.144 | 0.291 | |
| C22:0 | 15.0±6.54 | 14.4±3.24 | 0.569 |  | 0.0969±0.0414 | 0.0976±0.0283 | 0.516 | |
| C16:1 | 720±380 | 640±255 | 0.588 |  | 4.27±1.58 | 4.14±1.31 | 0.957 | |
| C18:1 | 4920±1370 | 4560±1040 | 0.123 |  | 30.0±3.29 | 29.9±3.28 | 0.818 | |
| C20:1 | 84.1±30.8 | 84.9±33.0 | 0.858 |  | 0.515±0.153 | 0.564±0.231 | 0.470 | |
| C16:2 | 11.2±5.24 | 9.41±2.91 | 0.023 |  | 0.0675±0.0202 | 0.0622±0.0167 | 0.057 | |
| C18:2 | 2630±876 | 2360±600 | 0.157 |  | 16.0±3.08 | 15.5±2.48 | 0.491 | |
| C18:3 | 442±236 | 341±159 | 0.022 |  | 2.71±1.37 | 2.21±0.862 | 0.057 | |
| C18:4 | 12.3±8.80 | 9.99±8.05 | 0.015 |  | 0.0746±0.0409 | 0.0693±0.0704 | 0.027 | |
| C20:2 | 54.0±14.7 | 51.4±12.9 | 0.360 |  | 0.332±0.0506 | 0.337±0.0503 | 0.535 | |
| C20:3 | 59.1±19.1 | 52.3±12.8 | 0.076 |  | 0.361±0.0680 | 0.344±0.0562 | 0.137 | |
| C20:4 | 151±57.4 | 136±43.0 | 0.156 |  | 0.923±0.229 | 0.901±0.246 | 0.491 | |
| C22:4 | 25.8±9.71 | 23.4±6.49 | 0.301 |  | 0.156±0.0295 | 0.154±0.0306 | 0.876 | |
| C22:5 | 101±56.1 | 85.6±41.6 | 0.048 |  | 0.604±0.221 | 0.563±0.242 | 0.079 | |
| C22:6 | 398±249 | 366±214 | 0.296 |  | 2.44±1.18 | 2.45±1.48 | 0.539 | |
| SFA | 6610±944 | 6410±770 | 0.285 |  | 41.6±5.23 | 42.8±5.10 | 0.280 | |
| MUFA | 5730±1700 | 5290±1260 | 0.135 |  | 34.8±4.22 | 34.6±4.18 | 0.993 | |
| PUFA | 3890±1220 | 3440±848 | 0.037 |  | 23.7±3.59 | 22.6±3.54 | 0.103 | |
| Data are represented as the mean ± SD and intensities was divided by 10^4^. *P*-values were calculated from Mann-Whitney *U*-tests. Abbreviations: AF, atrial fibrillation; SFA, saturated fatty acid; MUFA, monounsaturated fatty acid; PUFA, polyunsaturated fatty acid. | | | | | | | |  |
