# Supplementary figures and images for: Lipidomic profiling reveals free fatty acid alterations in plasma from patients with atrial fibrillation
Source: PLoS One. 2018 May 3;13(5):e0196709. doi: 10.1371/journal.pone.0196709 (PMC5933795; doi:10.1371/journal.pone.0196709)

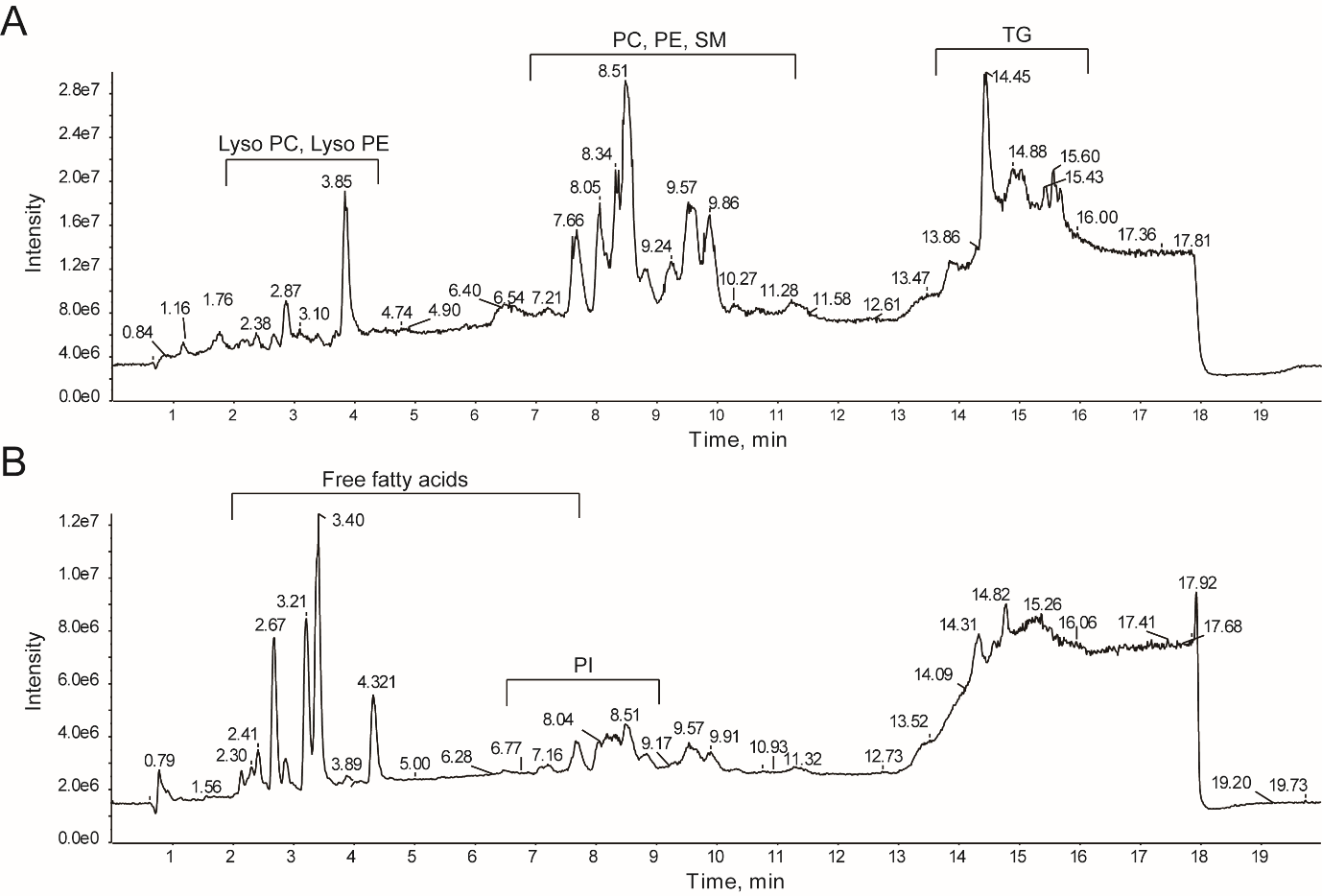

Supplement: S1 Fig — (A) Positive ion mode and (B) negative ion mode. (TIF) [file pone.0196709.s003.tif]

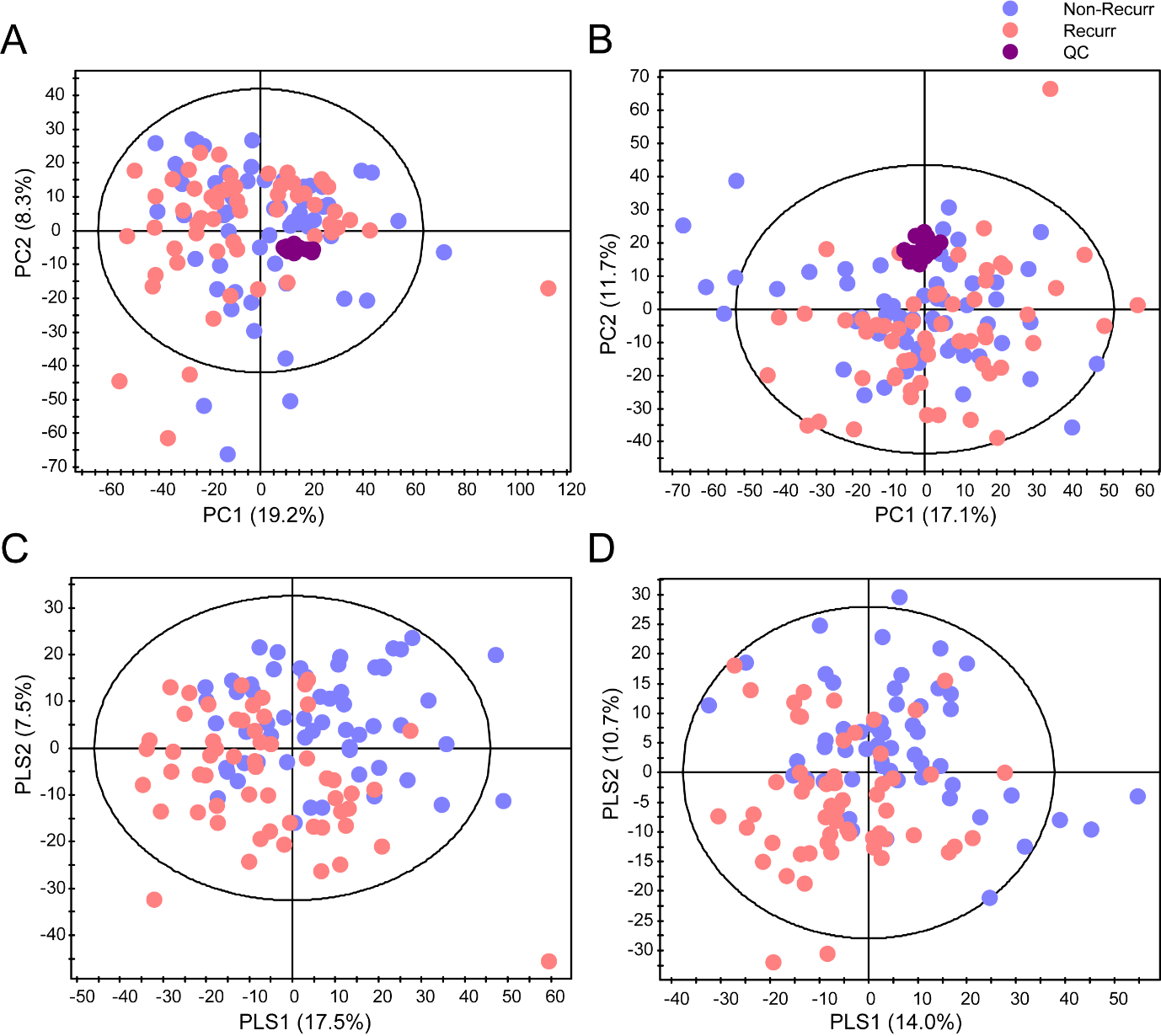

Supplement: S2 Fig — PCA score plots in positive (A; R2X = 0.708, Q2 = 0.526) and negative ion mode (B; R2X = 0.705, Q2 = 0.509). Each ellipse was given by Hotelling’s T2 (0.95). (TIF) [file pone.0196709.s004.tif]
